# FID-YOLO: A pedestrian detection model integrating multispectral information in complex environments

**DOI:** 10.1371/journal.pone.0342054

**Published:** 2026-03-05

**Authors:** Di Yang, Xilong Zhang, Peng Wang

**Affiliations:** 1 School of Computer Science and Technology, Changchun University of Science and Technology, Changchun, China; 2 Jilin Provincial Joint Key Laboratory of Big Data Science and Engineering, Changchun, China; 3 Jilin Provincial Science and Technology Innovation Center of Network Database Application Software, Changchun, China; Northwestern Polytechnical University, CHINA

## Abstract

The advancement of pedestrian detection technology is of great importance for various applications such as intelligent driving, object tracking, and robot navigation. Many studies in this field have demonstrated that image quality significantly contributes to the precision of detection. However, unexpected factors such as adverse weather, occlusions, and scale variations, which extremely weaken the main features of the detected objects, leading to a decrease in detection accuracy. To address these problems, we propose a Feature-enriched Image Detection-YOLO (FID-YOLO), to improve pedestrian detection performance in complex environments by integrating visible and infrared light information. Specifically, we design an illumination-aware image fusion module for visible and infrared image information fusion to generate a new image within more information to enrich pedestrian features. Then, a cascaded feature aggregation module using reparameterization and channel shuffle is introduced to enhance the model’s understanding and generalization capabilities for complex scenes. Furthermore, we exploit a scale-adaptive feature detection head for YOLO detector, which solves the problem of detecting small objects at varying object scales. Experiments on M3FD and LLVIP datasets demonstrate that FID-YOLO outperforms the benchmark models in pedestrian detection. Additionally, we validate the indispensability of each proposed module through ablation experiments.

## Introduction

Accurately detecting and localizing pedestrians in complex environments is a critical task in computer vision. The frequent occurrence of pedestrian traffic accidents has brought great pressure to traffic safety, high-precision pedestrian detection to meet the need in applications such as autonomous driving systems and road safety monitoring, is highly desiderated [1]. With the continuous advancement in pedestrian detection technology, it significantly contributes to the reduction of traffic accidents and the enhancement of pedestrian safety, which in turn increases road traffic efficiency and promotes the development of intelligent transportation systems.

**Fig 1:**
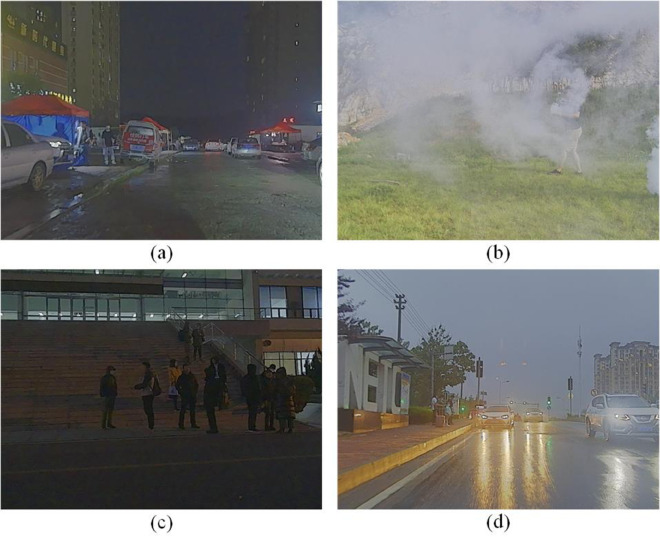
Objects in complex environments (a) Nighttime small pedestrians detection (b) Smoky condition pedestrians (c) Nighttime occlusion detection (d) Rainy occluded object detection.

The quality of the images directly impacts the accuracy of pedestrian detection [2]. The captured images are often affected by complex environmental factors, such as occlusions and small objects in low illumination conditions. Fig 1 respectively shows pedestrians under various challenging conditions: at night, in smoky environments, with mutual occlusions, and in low-resolution images captured on rainy days. Such complex conditions significantly reduce the accuracy of pedestrian detection. Therefore, optimization of pedestrian detection in these challenging environments is extremely crucial for enhancement of pedestrian detection performance.

Detecting pedestrians within dynamic and changeable environments is a major challenge in the field of computer vision. Over the past few decades, a variety of methods is employed to conduct diverse studies on object detection. Prior to the end-to-end detection, many researches constructed by traditional vision measurement, such as histogram of oriented gradients (HOG) [3,4], local binary pattern (LBP) [5] and aggregate channel features (ACF) [6], these methods based on hand crafted features and classifiers to detect objects. Although widely adopted in the early stages, however, the predictive accuracy critically depends on the quality of these hand-designed features. This often leads to poor generalization of the models when dealing with pedestrian objects in complex environment.

Currently, the advantages of deep learning are becoming increasingly prominent. Compared to traditional vision measurement methods, deep learning has proven highly effective at capturing the complex features and variable behavior of pedestrians, resulting in widespread use and significant benefits. Specifically, pedestrian detection approaches can be categorized into one-stage and two-stage methods. One-stage object detection methods simultaneously detect and classify objects in an image by using anchor boxes to predict bounding boxes and class labels directly. These methods have advantages in high efficiency but may sacrifice accuracy. Examples include SSD [7,8], YOLO [9–11], and RetinaNet [12]. Two-stage object detection methods first generate a set of proposals, typically using a Region Proposal Network (RPN), and then refine these proposals to predict final bounding boxes and class labels. Although these methods generally achieve higher accuracy, they often involve longer processing times. Such as R-CNN [13], SPPNet [14], Faster R-CNN [15], Mask R-CNN [16]. One-stage and two-stage methods each offer distinct trade-offs between speed and accuracy, making them applied to different application scenarios. On the basis of the foregoing, researchers have further improved object detection accuracy by incorporating auxiliary technologies, most previous researches have utilized image fusion [17], feature pyramid networks [18–21], transfer learning [22–24], and attention mechanisms [25,26] to augment deep learning methods in addressing the challenges of feature information loss, modality imbalance, and position offset. Specifically, the feature pyramid network is constructed by a multi-scale feature representation integrating feature maps at various levels, effectively enhancing the ability to detect pedestrians of different sizes. The top-down pathway and lateral connections ensure an effective integration of high-level semantic information with low-level details, improving the precision of object localization. While transfer learning applies knowledge from pre-trained models to related but different tasks, accelerating model convergence and enhancing robustness. Additionally, the attention mechanism in pedestrian detection enhances the model’s ability to focus on critical features, thereby boosting its detection performance. Despite significant progress in pedestrian detection, there are still some challenges that deserve to be emphasized:

Firstly, image fusion could lead to information loss and color distortion, which may prevent‘ accurately reflect details and features of the original images. Meanwhile, due to the low resolution of pedestrian images in low light conditions, the edges of pedestrian objects become blurred. Therefore, it is significant to develop methods that effectively restore object boundaries and improve overall image quality. Secondly, occlusion in pedestrian detection could occur in two forms: one is occlusion between pedestrians, and the other is occlusion of pedestrians by other objects. These two types of occlusions have varying degrees of impact on the detector’s ability to detect targets, potentially leading to missed detection and reduced accuracy. Moreover, the distance between pedestrians and the camera could cause changes in pedestrian scale, low-resolution pedestrians at smaller scales are more prone to missed detection. Thus, improving detection accuracy for small-scale pedestrians is crucial.

To overcome these limitations, we propose a feature-enriched image detection-YOLO (FID-YOLO), a pedestrian detection framework. This model adopts the YOLO architecture to improve image quality, and minimize detection errors. By integrating visible and infrared light information, the proposed method outperforms traditional detection techniques that rely on a single light source, thereby improving the precision of pedestrian detection. The main contributions of this work are summarized as follows:

- An illumination-aware image fusion module is designed to integrate infrared and visible light information, generating a composite image with deep semantic information and fine-grained details. It employs spatial and channel attention mechanisms to focus on crucial details in both visible and infrared images during the fusion process, achieving a more comprehensive pedestrian feature representation.
- A cascaded feature aggregation module is designed for multi-scale feature fusion, distributing the fused features across different scales. This module enables the model to capture object information more comprehensively, including partially occluded instances. By integrating features from multiple scales, the module improves the model’s ability to recognize and localize occluded objects, ensuring robust detection even when visibility is compromised.
- A scale-adaptive feature detection head is proposed to capture object features at different scales more effectively, enhancing the detection of small objects. This module achieves task alignment in both label assignment and detection by using a feature extractor that learns interactive features from multiple convolutional layers. The joint features improve inter-task interaction.
- The proposed method is validated on the LLVIP and M3FD datasets. Ablation experiments are also conducted to demonstrate the effectiveness of the proposed modules.

## Related works

### Occlusion in pedestrian detection

Among numerous visual recognition tasks, occluded object detection is particularly critical, as it involves effectively recognizing and tracking objects that are partially obscured by others. Li et al. [27] addressed the issue of feature confusion caused by occlusions in UAV imagery by proposing an occlusion estimation module (OEM) for precise occlusion localization. The author implemented occlusion-guided detection through multi-task interactions, effectively resolving the occlusion challenges in object detection for UAV images. Hao [28] proposed an anchor-free infrared pedestrian detection algorithm that enhances the detection performance of multiscale and partially occluded objects by designing a cross-scale feature fusion module. Additionally, a hierarchical attention mapping module is constructed to increase the significance of pedestrian features in complex environments while suppressing background information. Shi et al. [29] proposed a Global-Local Awareness Detector (GLA-D) to extract scale variance feature information from input frames, addressing the scale variations of objects moving in the scene and the frequent occlusions caused by complex scenes. Additionally, they introduced Occlusion Awareness Data Association (OADA), which uses different metrics for high- and low-scoring detection frames to alleviate occlusion issues in tracking scenarios.

### Adverse weather pedestrian detection

Pedestrian detection in adverse weather conditions faces challenges such as poor image quality, uneven lighting, and low contrast. These factors could lead to a decline in detection performance and increase the risk of traffic accidents. To address these issues, researchers have conducted extensive studies. Liu et al. [30] employed six differentiable filters to automatically adjust parameters based on the brightness and weather information from the input image. These adjustments aim to mitigate adverse factors affecting the image and restore its underlying content, thereby enhancing detection performance. Additionally, a mixed dataset comprising normal and low-quality images was used to ensure that IA-YOLO can adaptively handle varying weather conditions. Luo et al. [31,32] addressed the challenge of degraded image quality in adverse weather conditions impacting object detection accuracy. Using the LaLM method, which enhances precision by minimizing the discrepancy between degraded and clean images at the prediction level rather than the image level itself, the researchers achieved superior detection accuracy and inference speed in experimental evaluations on foggy, rainy, and low-light scenarios.

### Small object detection

Small object detection is an advanced field in computer vision that focuses on identifying and analyzing small objects in images with limited details. Qin et al. [33] separate the target from the background based on the diversity of morphological features and consider the continuity of target motion in the time domain by using the RX filter to extract the target trajectory in random projection. Experiments on various cluttered background sequences validate the proposed method’s detection capability in the field of infrared small target detection. Wang et al. [34] developed a new lightweight network model named HV-YOLOv8, which enhances the accuracy of small object detection by incorporating residual structures within the convolutional modules and by introducing the variety of view group shuffle cross stage partial network (VOV-GSCSP) module. This approach significantly reduces the number of parameters and computational requirements. Moreover, relying solely on visible light often provides insufficient information for pedestrian detection, such as nighttime and small-scale objects. Cao et al. [35] introduce the LG-FAPF network, an end-to-end system that leverages locality-guided cross-modal feature aggregation and pixel-level fusion to learn robust pedestrian representations. The network effectively encodes local responses and mutual cues from various instances into a unified descriptor and fuses visible and thermal information for accurate detection. Wei et al. [36] combined UNet and YOLO for visible and infrared fusion, performing object detection by sharing visible light information. These methods enhance detection accuracy through image fusion techniques. However, the generated pseudo-color images have some stain-like areas that can block the field of view, which may have impact on the accuracy of object detection.

Most of the researches mentioned above have been proposed to improve detection accuracy, it is still challenging under complex and adverse weather conditions. Therefore, integrating visible and infrared light for object detection is anticipated to be a major focus in the field.

## Methodology

**Fig 2:**
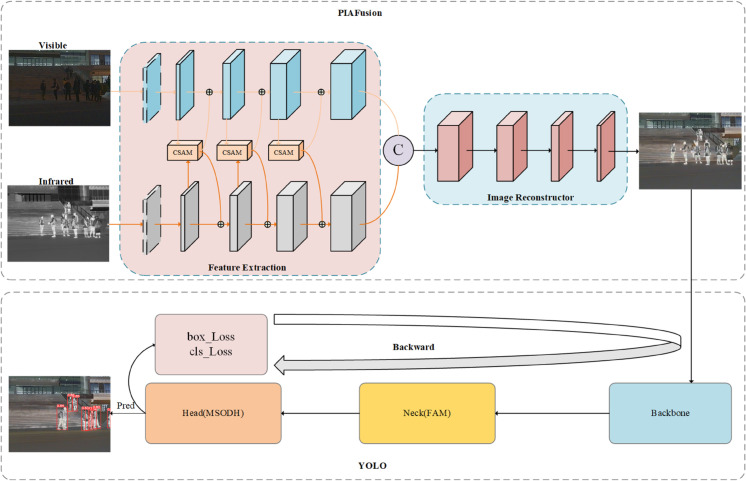
The structure and workflow of the proposed FID-YOLO.

In order to address the increasingly challenging traffic environments, we propose a pedestrian detection model, as depicted in Fig 2. The model consists of two main parts: the illumination-aware image fusion module and the object detection module.

The illumination-aware image fusion module, which builds upon the progressive infrared and visible images fusion network to generate a fusion image within more feature information. It involves replacing the channel attention module in the cross-modality differential aware fusion (CMDAF) with the channel and spatial attention module (CSAM). This adjustment enables the model to more effectively capture main features while enhancing its ability to suppress background noise, aim to improve prediction accuracy and generalization capabilities. Additionally, the loss function has been optimized by replacing the original L1 norm with the Huber loss, which enhances training efficiency and stability. The image fusion process produces a single image with complementary information, enhancing pedestrian features for more effective pedestrian detection.

The object detection module is built on the YOLOv8 network, with a cascaded feature aggregation and extraction (CFAE) module integrated between the backbone and neck to optimize feature fusion across scales. The CFAE module combines shallow and deep features through channel shuffling, improving feature integration without adding unnecessary complexity or resource demands. A reparameterization operation is introduced to reduce computational and memory costs during inference. Additionally, a scale-adaptive feature detection head (SAFDH) is designed to use shared convolutions, reducing parameters while enabling the feature extractor to learn interactive features across tasks, boosting detection accuracy in complex environments.

**Fig 3:**
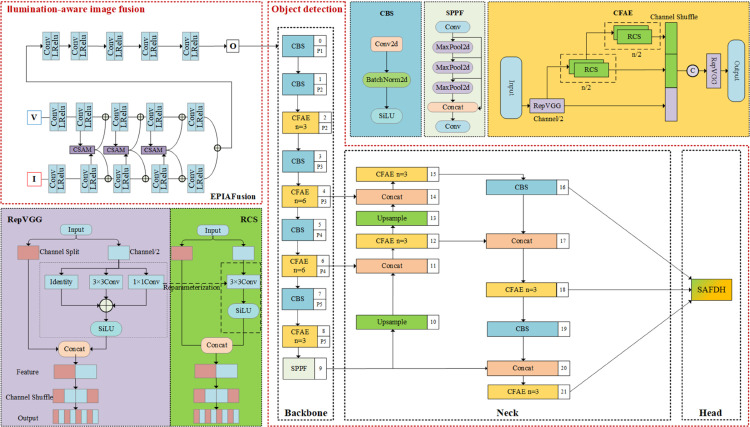
Overall structure of infrared and visible light fusion object detection network.

To be specific, Fig 3 is a detailed expansion of Fig 2, offering a more comprehensive illustration of the overall architecture of the pedestrian network. First of all, the progressive infrared and visible images as inputs are processed by the illumination-aware image fusion module to extract both deep and shallow features at multiple scales, then fused by differential computing to generate a fusion image with complementary features. Subsequently, the fusion image will be fed into object detection module, which consists of the backbone, the neck, and the head networks. In particular, the generated fusion image is inputted into backbone to learn multi-modal feature representations. Then, the obtained implicit representation is passed to the neck network for feature refinement and enhancement. Finally, the head network conducts the final pedestrian detection, outputting bounding boxes and classification results in complex environments.

### Illumination-aware image fusion module

Accurate detection of pedestrian objects primarily depends on the robustness of image features. However, existing methods mainly perform object detection based on single light source images, which can lead to blurred feature representations under low-light conditions, adverse weather, or occlusion. To preserve more comprehensive image features, we construct an illumination-aware image fusion module that integrates visible and infrared images. Visible images contain rich texture and color information, whereas infrared images emphasize thermal targets. By connecting these two feature manifolds, the module enables complementary feature extraction, enhancing the distinction between background and object information while suppressing noise.

The proposed module employs an illumination-aware sub-network to incorporate environmental illumination as a prior for fusion, inspired by PIAFusion [37]. However, a critical theoretical limitation of PIAFusion is its reliance solely on channel attention mechanisms within its Cross-Modality Differential Aware Fusion (CMDAF). While channel attention effectively recalibrates the importance of different feature maps, it ignores the spatial distribution of features. In pedestrian detection, spatial details—such as limb positioning and edges—are crucial. To address this spatial information loss, we propose the Enhanced Progressive Infrared and Visible Image Fusion Network (EPIAFusion). The core innovation of EPIAFusion is the integration of a Channel and Spatial Attention Module (CSAM) into the differential fusion process. By simultaneously modeling inter-channel dependencies and inter-spatial relationships, CSAM allows the network to focus on the semantic content of the pedestrian while preserving the geometric details of the scene.

**Fig 4:**
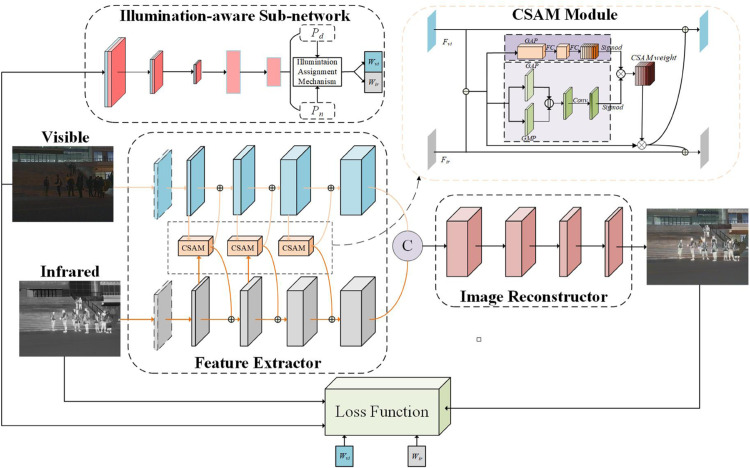
The structure of EPIAFusion module.

In EPIAFusion, a feature encoder is used to derive advanced features from both visible light and infrared light images, *I*_*ir*_ represented as follows: $I_{vi}$ represents the visible light image, represents the infrared light image, $F_{vi}$ and *F*_*ir*_ represent the visible light features and infrared light features respectively, and represent the feature extraction module. Fig 4 illustrates the structure of EPIAFusion. In EPIAFusion, a feature encoder is used to derive advanced features from both visible light and infrared light images, *I*_*ir*_ represented as follows: $I_{vi}$ represents the visible light image, represents the infrared light image, $F_{vi}$ and *F*_*ir*_ represent the visible light features and infrared light features respectively, and represent the feature extraction module.

$$\left\{F_{vi},F_{ir}\right\}=\left\{E_{F}\left(I_{vi}\right),E_{F}\left(I_{ir}\right)\right\}$$ (1)

To effectively fuse this differential information, the CSAM is applied as a compensation mechanism. Theoretically, simple addition of features introduces noise; CSAM acts as a dynamic filter. Eq (2) elucidates the principle of differential decomposition, where $F_{vi}^{i}$ and $F_{ir}^{i}$ denotes the features extracted by the i-th convolutional layer. By employing the channel and spatial attention module (CSAM) to compensate, the differential information between visible and infrared light can be effectively reconciled.

$$\begin{array}{c} F_{vi}^{i}=\frac{F_{vi}^{i}+F_{vi}^{i}}{2}+\frac{F_{ir}^{i}-F_{ir}^{i}}{2}=\frac{F_{vi}^{i}+F_{ir}^{i}}{2}+\frac{F_{vi}^{i}-F_{ir}^{i}}{2}\\ F_{ir}^{i}=\frac{F_{ir}^{i}+F_{ir}^{i}}{2}+\frac{F_{vi}^{i}-F_{vi}^{i}}{2}=\frac{F_{ir}^{i}+F_{vi}^{i}}{2}+\frac{F_{ir}^{i}-F_{vi}^{i}}{2} \end{array}$$ (2)

Therefore, the CSAM module is defined as shown in Eq (3), where ⊕ represents element-wise summation, ⊙ represents channel-wise multiplication, *δ*, *GAP* and *GMP* respectively denote the sigmoid function, global average pooling, and global max pooling operations. By applying the sigmoid function to scale the generated weights to the range [0, 1], the results from combining channel attention and spatial attention are added as modality compensation information to the original features, that mitigates the impact of background noise to outstand the main features for object detection.

To assign different weights to images for day and night condition, the light perception process in the light perception subnetwork is defined as follows: *P*_*d*_ and *P*_*n*_ represent the likelihood of whether an image is taken during the day or night. Since visible light images provide more detailed information about lighting conditions, the illumination probability is derived from these visible light images. This light probability is then used to calculate the light perception weights $w_{vi}$ for visible light and *w*_*ir*_ for infrared.

$${\hat{F}}_{vi}^{i}=F_{ir}^{i}⊕\delta \left(GAP\left(F_{1}\right)\right)⊙\delta \left(Conv\left[GAP\left({\bar{F}}_{1}\right),GMP\left({\hat{F}}_{1}\right)\right]\right)⊙F_{1}\;{\hat{F}}_{ir}^{i}=F_{ir}^{i}⊕\delta \left(GAP\left(F_{2}\right)\right)⊙\delta \left(Conv\left[GAP\left({\bar{F}}_{2}\right),GMP\left({\hat{F}}_{2}\right)\right]\right)⊙F_{2}$$ (3)

$$F_{1}=F_{vi}^{i}-F_{ir}^{i}\;F_{2}=F_{ir}^{i}-F_{vi}^{i}$$ (4)

$$\{P_{d},P_{n}\}=N_{IA}\left(I_{vi}\right)$$ (5)

$$W_{ir}=\frac{P_{n}}{P_{d}+P_{n}}\;W_{vi}=\frac{P_{d}}{P_{d}+P_{n}}\;$$ (6)

Standard fusion networks often rely on L1 or L2 norms, both of which exhibit inherent limitations. The L2 norm is highly sensitive to outliers and tends to produce blurred edges, whereas the L1 norm may lead to unstable gradient updates when the error approaches zero. To improve training stability and accelerate convergence, we replace the original L1 norm with the Huber loss. The Huber loss offers a robust trade-off by exhibiting quadratic behavior for small errors, which ensures smooth differentiability and fine-grained parameter updates, while transitioning to linear behavior for large errors, thereby reducing sensitivity to outliers such as extreme pixel intensity variations. The loss function for the image fusion network is formulated as a weighted combination of illumination loss, auxiliary intensity loss, and texture detail loss, as shown in Eq (4):

$${ℒ}_{fusion}=\lambda_{1}\cdot {ℒ}_{illum}+\lambda_{2}\cdot {ℒ}_{aux}+\lambda_{3}\cdot {ℒ}_{texture}$$ (7)

The improved light perception loss calculation formula is shown in Eq (8), where ${ℒ}_{\text{Huber}}^{vi}$ and ${ℒ}_{\text{Huber}}^{ir}$ represent the intensity losses for visible and infrared light, respectively.

$${ℒ}_{\text{illum}}=W_{vi}\cdot {ℒ}_{\text{Huber}}^{vi}+W_{ir}\cdot {ℒ}_{\text{Huber}}^{ir}$$ (8)

The intensity loss can balance the differences in pixel values between the pre-fusion and post-fusion images. Therefore, the intensity loss is defined as follows:

$$\begin{array}{c} {ℒ}_{\text{Huber}}^{\text{ir}}=\left\{ \begin{array}{cc} \frac{1}{2}{\left(I_{f}-I_{ir}\right)}^{2} & \text{for }|I_{f}-I_{ir}{|}_{2}\leq \delta \\ \delta \cdot \left(||I_{f}-I_{ir}|{|}_{2}-\frac{1}{2}\delta^{2}\right) & \text{otherwise} \end{array}\right.\\ {ℒ}_{\text{Huber}}^{vi}=\left\{ \begin{array}{cc} \frac{1}{2}{\left(I_{f}-I_{vi}\right)}^{2} & \text{for }|I_{f}-I_{vi}{|}_{2}\leq \delta \\ \delta \cdot \left(||I_{f}-I_{vi}|{|}_{2}-\frac{1}{2}\delta^{2}\right) & \text{otherwise} \end{array}\right. \end{array}$$ (9)

In the context of defining the intensity loss within an image fusion network, the terms $||I_{f}-I_{vi}|{|}_{2}$ and $||I_{f}-I_{ir}|{|}_{2}$ represent the Euclidean distances between the fused image and the respective visible light image and infrared image. The parameter *δ* is the threshold for the Huber loss function.

Relying solely on light intensity is insufficient to maintain an optimal intensity distribution in the fused image. Therefore, an auxiliary intensity loss is defined as follows:

$${ℒ}_{aux}=\frac{1}{HW}||I_{f}-\max\left(I_{ir},I_{vi}\right)|{|}_{1}$$ (10)

To preserve detailed textures in the fused image, a texture detail loss is definded. The gradient operator ∇ is used to capture the texture information within the image, where the gradients are computed using the Sobel operator.

$${ℒ}_{texture}=\frac{1}{HW}|||\nabla I_{f}|-max\left(|\nabla I_{vi}|,|\nabla I_{ir}|\right)|{|}_{1}$$ (11)

### Cascaded feature aggregation and extraction module

**Fig 5:**
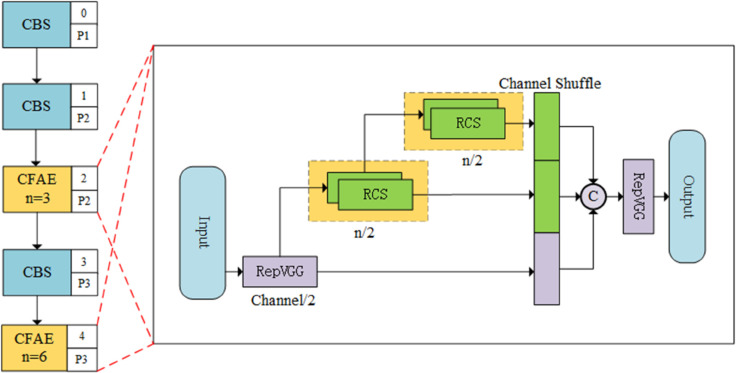
The structure of the CFAE module.

To address the feature dilution effect caused by heterogeneous interference in multi-spectral fusion and enhance the capture of pedestrian semantics in complex backgrounds, the Cascaded Feature Aggregation and Extraction (CFAE) module is designed and constructed, as illustrated in Fig 5. The module combines structural reparameterization with a multi-level feature aggregation mechanism, achieving a balance between representational expression and computational efficiency by equilibrating deep semantic extraction and the preservation of original spatial features.

**Fig 6:**
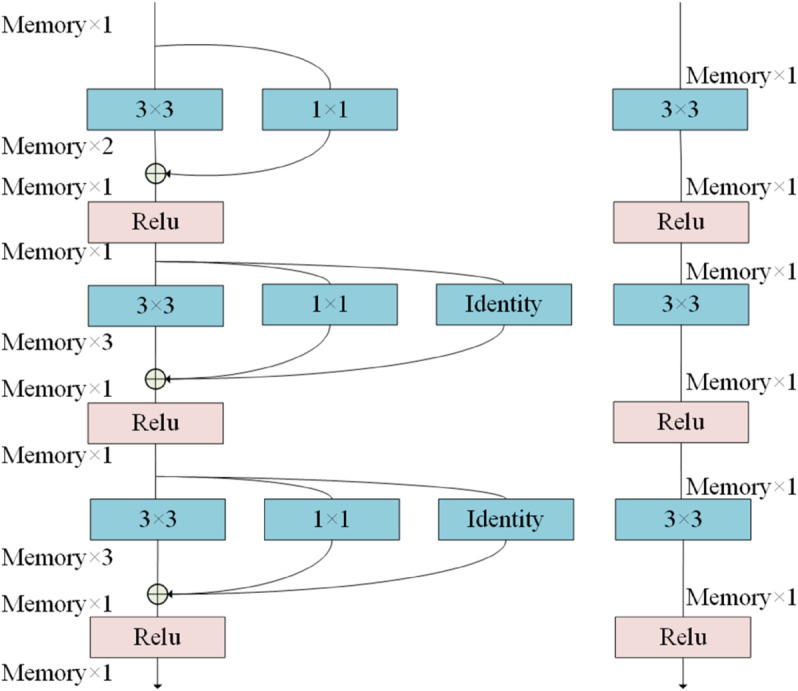
The structure of RepVGG module.

Within the CFAE module, the RepVGG unit serves as the fundamental feature extraction operator. As depicted in Fig 6, this unit utilizes a multi-branch topology during the training phase to capture diverse spatial gradients and smooth the loss landscape of complex multi-spectral data. Its training output, *F*_*train*_, is defined as follows:

$$F_{train}(x)=\text{BN}(x*K_{3})+\text{BN}(x*K_{1})+\text{BN}(x)$$ (12)

where *K*_3_ and *K*_1_ represent the kernel parameters for the 3×3 and 1×1 convolutions, respectively. In the inference phase, these branches are collapsed into an equivalent singular kernel, $K_{eq}=f_{rep}(K_{3},K_{1},I)$, through structural reparameterization. This transformation ensures semantic abstraction while maintaining *O*(1) memory complexity, addressing the computational burden of multi-spectral models during deployment.

**Fig 7:**
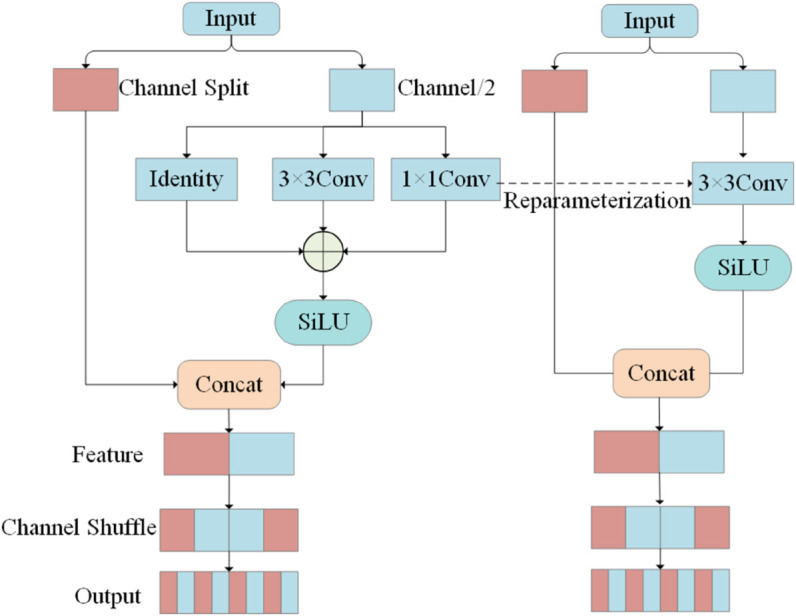
The structure of RCS module.

CFAE integrates the Shuffle RepVGG (SR) structure, which corresponds to the cascaded RCS component shown in Fig 7. The SR unit employs a channel-splitting strategy, partitioning the input feature *X* into a primary stream *X*_*prim*_ and an identity stream *X*_*cons*_:

$$F_{SR}=\text{Shuffle}\left(\text{Concat}(X_{cons},\Phi_{Rep}(X_{prim}))\right)$$ (13)

where $\Phi_{Rep}$ denotes the RepVGG operator applied to the primary path. This configuration establishes a controlled residual path for deep networks, ensuring that the raw spatial information of pedestrian targets contained in the identity stream *X*_*cons*_ is preserved during the cascading process. Subsequent channel shuffle operations, as detailed in Fig 7, complete the cross-channel information interaction between the disparate streams.

Furthermore, as shown in Fig 5, CFAE adopts a cascaded One-Shot Aggregation (OSA) mechanism. It integrates the initial projection feature *x*_1_ and the successive outputs of the SR units, *x*_2_ and *x*_3_, through a concatenation operation:

$$F_{CFAE}=\Phi_{out}(\text{Concat}(x_{1},x_{2},x_{3}))$$ (14)

where $\Phi_{out}$ represents the final projection operator. This cascaded design constructs an ensemble of multi-scale receptive fields capable of aggregating implicit semantic features from varying depths. This mechanism improves the reconstruction of partially occluded pedestrians and limits inference-time computational overhead through reparameterization.

### Scale-adaptive feature detection head

**Fig 8:**
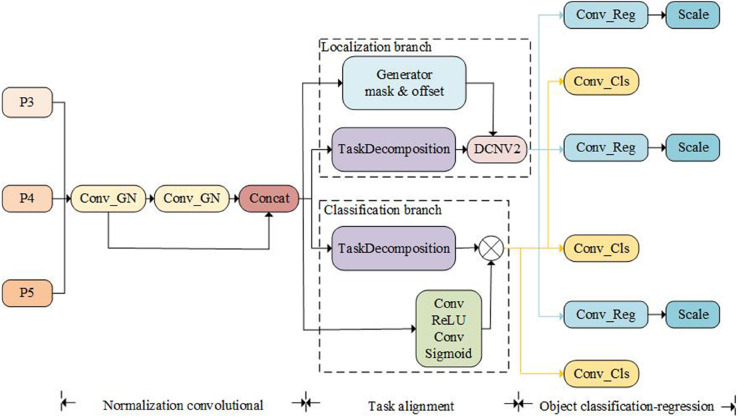
The structure of SAFDH module.

To overcome the inherent limitations of rigid feature extraction in multi-scale pedestrian detection, we propose a Scale-Adaptive Feature Detection Head (SAFDH), as illustrated in Fig 8. Unlike conventional detection heads that decouple classification and localization into parallel but homogeneous branches, SAFDH is formulated from a task–scale interaction perspective. Specifically, it explicitly models how object scale and task objectives jointly influence feature representation, which is particularly critical under multi-spectral feature fusion where spatial inconsistency and scale ambiguity frequently coexist.

A key design principle of SAFDH is to establish a stable and scale-consistent shared representation prior to task specialization. To this end, we introduce a Shared Normalization Convolution (SNC) module, implemented as share_conv, which combines shared convolution with Group Normalization (GN) [38]. From a theoretical standpoint, GN mitigates feature distribution shifts caused by illumination and modality variations, while parameter sharing constrains both tasks to operate on a unified semantic manifold. This design not only reduces computational redundancy but also provides a well-conditioned feature space for subsequent task-aware decomposition.

Recent studies, such as TOOD [39], have demonstrated that modeling task interaction can improve detection performance by aligning classification and localization features within a shared interaction space.However, directly enforcing task alignment may obscure the fundamentally different optimization requirements of the two tasks, especially in multi-scale pedestrian detection scenarios where localization is highly scale-sensitive while classification emphasizes semantic consistency. Instead of directly aligning tasks through a single interaction space, SAFDH decomposes task learning into two scale-adaptive and asymmetric pathways. We construct two independent Adaptive Task Decomposition (ATD) branches, denoted as cls_decomp and reg_decomp, which dynamically reweight shared features conditioned on global contextual statistics. The task-specific transformation is formulated as

$$F_{\text{task}}=\Psi_{\text{ATD}}\left(F_{\text{shared}},\mathrm{Pool}\left(F_{\text{shared}}\right)\right),$$ (15)

where $F_{\text{shared}}$ represents the SNC output, and Pool(·) encodes global scale information. This formulation reflects the inherently different optimization requirements of localization and classification, in which localization emphasizes scale-dependent geometric boundaries, whereas classification focuses on scale-invariant semantic discrimination. By decoupling these objectives through ATD, SAFDH reduces task interference and enables scale-aware feature specialization, rather than enforcing uniform task alignment.

Furthermore, SAFDH introduces an asymmetric spatial alignment strategy tailored to the geometric nature of pedestrian localization. An Offset and Mask Mechanism (OMM) is employed to generate pixel-wise offsets and modulation masks, which guide a Dynamic Deformable Convolutional Network v2 (DyDCNv2) [40] exclusively in the regression branch. Spatial misalignment has a more pronounced impact on localization accuracy, whereas introducing excessive spatial deformation in the classification branch may undermine semantic consistency.

## Experiment setup and result analysis

### Dataset and model training details

In subsequent experiments, we validate the FID-YOLO model using two public datasets: M3FD [41] and LLVIP [42].

(1) M3FD Dataset: The M3FD Dataset, The M3FD Dataset, created by Peng Cheng Laboratory, contains 4,200 pairs of aligned infrared and visible light images across four challenging scenarios: daytime, overcast, nighttime, and occlusion. It includes 33,603 object annotations in six categories (People, Car, Bus, Motorcycle, Truck, and Lamp). The dataset’s diversity in pixel variations, lighting, seasons, and weather makes it valuable for training and evaluating object detection in fused images.

(2) LLVIP Dataset: The LLVIP Dataset focuses on low-light object detection with 15,488 pairs of visible and infrared images (30,976 images in total) from 24 nighttime and 2 daytime scenes. It provides temporally and spatially aligned image pairs, with annotations for objects detectable in infrared under dim lighting, making it essential for low-light detection research.

The experimental configuration is detailed in Table 1. For the experiments conducted in this study, the Stochastic Gradient Descent (SGD) optimizer is employed to train the YOLO model with input images resized to 640×640 pixels. The batch size is configured at 32, with an initial learning rate of 0.01. 16 threads are allocated for data loading. Additionally, mosaic data augmentation, a technique used to increase the robustness of the model by augmenting the training data, is deactivated during the final ten epochs of the training regimen.

**Table 1:** Experimental environments and model parameter configuration.

Name	Version
Operating System	Ubuntu 22.04.4 LTS
CPU	13th Gen Intel(R) Core(TM) i9-13900K
GPU	NVIDIA GeForce RTX 4090 24G
Python	3.8
PyTorch	2.3.0
CUDA	12.1
Input size	640×640
Batch size	32
Initial learning rate	0.01
Final learning rate	0.1

### Evaluation metrics

We evaluate the effectiveness of the model on the M3FD and LLVIP datasets by a suite of commonly used performance metrics, including mean Average Precision (mAP) at an Intersection over Union (IoU) threshold of 0.5 (mAP@0.5), mean Average Precision across the IoU range from 0.5 to 0.95 (mAP@0.5-0.95), and the F1 score. Furthermore, this study evaluates the computational consumption of the model by examining the quantity of parameters.

### Experimental comparisons

We compare our model with the YOLO series, including YOLOv5n [43], YOLOv6n [44], YOLOv7-tiny [45], YOLOv8n [46], YOLOv8s [46], YOLO-MIF-n [47], and ICA-Fusion-n [48], RGBT-YOLO [49], DAMSDet [50], WaveMamba [51], LASFNet [52] on LLVIP and M3FD datasets to validate the performance of our model, in which considering the computational comsuption, we choose medium and small models within the YOLO series, where the ‘-n’, ‘-s’ and ‘-m’ respectively represent nano, small, and medium models.

In addition, to further evaluate the cross-dataset generalization capability of the proposed method, we conduct additional experiments on the WiderPerson dataset. WiderPerson is a representative dense pedestrian detection benchmark, whose images are primarily collected from the Internet and exhibit high scene complexity, including cluttered backgrounds and watermark interference. The dataset defines five categories for pedestrian-related objects: (1) pedestrians, (2) riders, (3) partially occluded persons, (4) person-like objects, and (5) dense crowd regions with indistinguishable individual boundaries. Since our objective is to assess generalization performance for pedestrian detection across datasets, categories 1–4 are unified as a single “pedestrian” class for training and evaluation, while category 5 is excluded due to the absence of clear instance-level annotations. This preprocessing strategy is consistent with common practices in existing dense pedestrian detection studies.

#### Comparative experiment on M3FD dataset.

**Fig 9:**
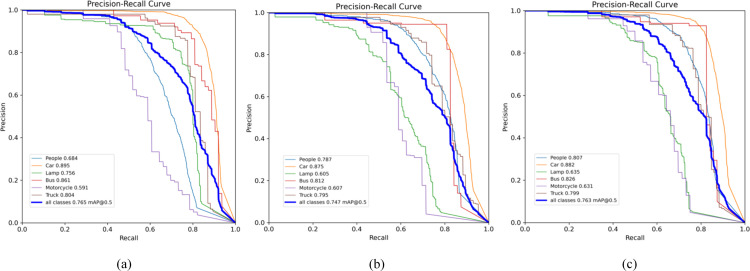
M3FD Dataset FID-YOLO and other models P-R curve comparison result.

**Fig 10:**
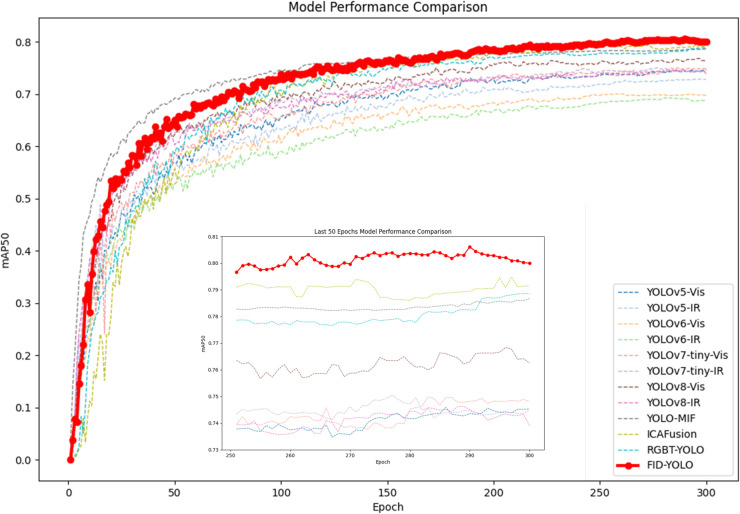
M3FD Dataset FID-YOLO and Other models mAP comparison result.

Table 2 shows the performance of the FID-YOLO-n model on the M3FD dataset compared to benchmark models. Our model outperforms YOLOv5-n, YOLOv6-n, YOLOv7-tiny, and YOLOv8-n in all metrics except for parameter scale. This indicates that the EPIAFusion module effectively enriches feature information, improving detection accuracy at the cost of computational resources. In visible light detection, our model achieves a pedestrian detection precision of 0.801, with a mAP@0.5 just 0.2% lower than YOLOv8-s. In infrared detection, despite a 2.4% lower precision, our mAP@0.5 improves by 2.6% compared to YOLOv8-s. YOLOv8-s achieves high precision by increasing parameter scale, but our model, with 27.7% fewer parameters, maintains a balance between detection accuracy and computational cost. Compared to the YOLO-MIF and ICAFusion models, designed for mixed light input, FID-YOLO achieves superior performance in most metrics, confirming the effectiveness of the EPIAFusion module for integrating visible and infrared images. Our model also achieves optimal mAP@0.5-0.95 and F1 scores. As observed from the Fig 9(a), 9(b), 9(c) represent the baseline model under visible light and infrared light, and the P-R curve of FID-YOLO, respectively. The area enclosed by the P-R curve and the coordinate axes is larger for FID-YOLO, indicating that, compared to the baseline model, FID-YOLO is better at balancing the identification of positive classes and the accuracy of predictions across different thresholds. Fig 10 shows FID-YOLO’s advantage in object detection in complex environments on the M3FD dataset, further validating the model’s effectiveness.

**Table 2:** The experimental effects of different models on the M3FD dataset.

Network architecture	Input type	Pedestrian	mAP@0.5	mAP@0.5-0.95	F1 score	Param(M)	GFLOPs
YOLOv5-n [43]	Visible	0.633	0.742	0.471	0.73	2.6	4.6
YOLOv5-n [43]	Infrared	0.790	0.728	0.457	0.72	2.6	
YOLOv6-n [44]	Visible	0.630	0.699	0.446	0.70	4.7	11.4
YOLOv6-n [44]	Infrared	0.758	0.690	0.441	0.69	4.7	
YOLOv7-tiny [45]	Visible	0.701	0.754	0.475	0.74	6.2	13.8
YOLOv7-tiny [45]	Infrared	0.753	0.701	0.455	0.70	6.2	
YOLOv8-n [46]	Visible	0.712	0.756	0.491	0.74	3.2	8.7
YOLOv8-n [46]	Infrared	0.787	0.747	0.488	0.74	3.2	
YOLOv8-s [46]	Visible	0.750	0.805	0.534	0.79	11.2	28.6
YOLOv8-s [46]	Infrared	0.825	0.779	0.506	0.77	11.2	
YOLO-MIF-n [47]	Mixed	0.792	0.786	0.514	0.77	3.3	12.3
ICAFusion-n [48]	Mixed	0.795	0.791	0.479	0.77	3.0	25.8
FID-YOLO-n (Ours)	Mixed	0.801	0.803	0.535	0.79	8.1	22.4

#### Comparative experiment on LLVIP dataset.

**Fig 11:**
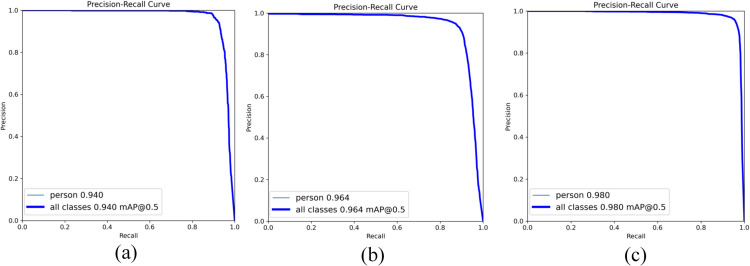
LLVIP dataset FID-YOLO and other models P-R Curve comparison result.

**Fig 12:**
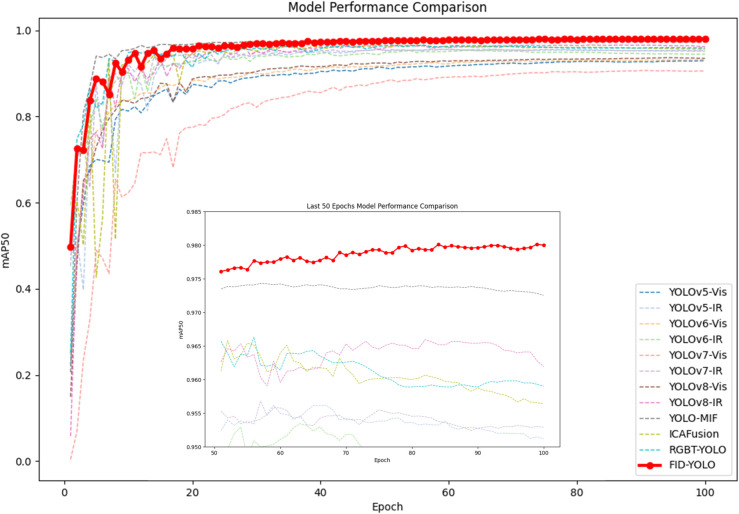
LLVIP dataset FID-YOLO and other models mAP comparison result.

Fig 11 shows the P-R curve comparison between FID-YOLO and the baseline model. The results indicate that FID-YOLO outperforms the baseline model in terms of precision and recall across different thresholds. Fig 12 and Table 3 show the results of benchmark models on the LLVIP dataset using inputs from different light sources. Pedestrian detection performance on infrared images significantly outperforms that of visible light images, as infrared images highlight object contours in low-light or dark conditions, while visible light often obscures object features. However, infrared images may miss environmental details. The integration of the EPIAFusion network improves detection performance, with a 4.5% increase in mAP@0.5 compared to YOLOv8 with visible light input, a 12.1% improvement in mAP@0.5-0.95, and a 3% increase in the F1 score. Compared to the infrared model, the improvement is 1.6% in mAP and 2% in F1 score.

**Table 3:** The experimental effects of different models on the LLVIP dataset.

Network architecture	Input type	mAP@0.5	mAP@0.5-0.95	F1 score	Param(M)	GFLOPs
YOLOv5-m [43]	Visible	0.929	0.544	0.89	20.8	64.2
	Infrared	0.953	0.615	0.90		
YOLOv6-m [44]	Visible	0.932	0.552	0.90	34.9	85.8
	Infrared	0.952	0.637	0.87		
YOLOv7-tiny [45]	Visible	0.905	0.467	0.87	6.0	13.8
	Infrared	0.958	0.633	0.91		
YOLOv8-m [46]	Visible	0.935	0.552	0.90	25.8	78.9
	Infrared	0.964	0.684	0.91		
YOLO-MIF-m [47]	Mixed	0.974	0.684	0.91	26.6	120.0
ICAFusion-m [48]	Mixed	0.765	0.618	0.94	34.5	96.32
RGBT-YOLO-m [49]	Mixed	0.959	0.600	0.92	33.4	109.82
DAMSDet [50]	Mixed	0.915	0.553	0.92	79.21	173.76
WaveMamba [51]	Mixed	0.983	0.660	0.95	69.1	128.91
LASFNet [52]	Mixed	0.974	0.676	0.94	7.7	-
FID-YOLO-m (Ours)	Mixed	0.980	0.673	0.93	28.3	89.39

In comparison with existing multispectral detection approaches, YOLO-MIF and WaveMamba achieve competitive performance in terms of mAP@0.5; however, they exhibit relatively lower mAP@0.5–0.95, suggesting limited localization accuracy under stricter IoU thresholds. DAMSDet demonstrates robust detection capability but incurs significantly higher computational cost, as reflected by its large parameter size and GFLOPs. LASFNet achieves a favorable balance between accuracy and model complexity, yet its overall performance remains slightly inferior to the proposed method under comprehensive evaluation metrics. Overall, FID-YOLO maintains strong detection accuracy while preserving moderate computational complexity, and consistently performs well across multiple evaluation metrics. These results demonstrate the effectiveness and robustness of the proposed approach for multispectral pedestrian detection under low-light conditions. In addition, Fig 12 illustrates the training curves of mAP@0.5 for different models on the LLVIP dataset, further confirming the stability and effectiveness of the proposed method.

#### Comparative generalization on WiderPerson dataset.

The WiderPerson dataset is a representative dense crowd pedestrian detection dataset. The images are primarily collected from the internet, and many samples contain visible watermarks, which increases the realism and complexity of the scenes. The dataset defines five annotated categories for pedestrian-related objects: normal pedestrians, cyclists, partially occluded human bodies, human-like objects and indistinguishable dense crowds. To align with the objective of evaluating cross-dataset generalization in pedestrian detection, we retained categories 1 to 4 and merged them into a single “pedestrian” class, while category 5 (dense crowd regions) was excluded because it does not provide clear individual-level bounding boxes and is therefore unsuitable for standard pedestrian detection. This preprocessing strategy is consistent with common practice in dense pedestrian detection studies.

Furthermore, since WiderPerson contains only visible-light images and does not involve multi-spectral or cross-modal image pairs, the experiments on this dataset were conducted using only the YOLO detection branch of our model. The proposed EPIAFusion module was not employed in this setting, as image fusion is not applicable. This setup enables a fair evaluation of the generalization capability of the learned detection representations under a significantly different data distribution.

The experimental results are summarized in Table 4. Compared with the baseline YOLOv8-n, our FID-YOLO achieves consistent improvements across multiple evaluation metrics. Specifically, FID-YOLO improves mAP@0.5 from 0.714 to 0.727, mAP@0.5–0.95 from 0.429 to 0.437, and F1-score from 0.68 to 0.69, while maintaining comparable precision and recall. Notably, these gains are achieved with only a modest increase in computational cost (GFLOPs and parameters), indicating that the performance improvement does not stem from excessive model complexity. These results demonstrate that the proposed feature representation and detection head learned from multi-spectral training data retain strong discriminative capability when transferred to a dense, single-modality pedestrian dataset with substantially different data characteristics. This confirms that the performance gains are not overfitted to M3FD or LLVIP, but instead reflect improved generalization ability under diverse pedestrian detection scenarios.

**Table 4:** Cross-dataset generalization performance on the WiderPerson dataset.

Network architecture	mAP@0.5	mAP@0.5-0.95	F1	Precision	Recall	GFLOPs	IT	Param(M)
YOLOv8-n	0.714	0.429	0.68	0.78	0.61	8.2	3.7	3.1
FID-YOLO-n	0.727	0.437	0.69	0.78	0.63	8.7	3.9	3.9

### Ablation experiments

This study conducts ablation experiments on both the M3FD and LLVIP datasets using YOLOv8 as the base model to assess the contribution of each module within FID-YOLO. The findings from these ablation studies are delineated in Tables 5 and 6, which provide a comprehensive view of the impact of each module on the overall performance of the FID-YOLO model. Tables 7 and 8 present the ablation results for the CSAM components and CFAE configuration choices on the M3FD and LLVIP datasets, respectively. The CSAM module consistently improves mAP compared to the baseline CMADF, indicating that integrating channel and spatial attention helps the fusion network better preserve both semantic and geometric information. The CFAE and SAFDH modules provide additional gains over using only RepVGG or TOOD, validating their effectiveness in multi-scale feature extraction and task-specific feature decomposition. Across both datasets, combining CSAM with the downstream CFAE and SAFDH modules yields the best performance, demonstrating that each component contributes meaningfully to the overall accuracy.

**Table 5:** M3FD ablation experiment results.

Baseline	PIAFusion	EPIAFusion	CFAE	SAFDH	mAP@0.5	mAP@0.5-0.95	F1 score
					0.712	0.491	0.74
	✓				0.765	0.493	0.76
		✓			0.774	0.501	0.77
			✓		0.735	0.500	0.76
YOLOv8n				✓	0.729	0.512	0.76
		✓	✓		0.791	0.520	0.77
			✓	✓	0.741	0.524	0.78
		✓		✓	0.799	0.522	0.79
		✓	✓	✓	0.801	0.535	0.79

**Table 6:** LLVIP ablation experiment results.

Baseline	PIAFusion	EPIAFusion	CFAE	SAFDH	mAP@0.5	mAP@0.5-0.95	F1 score
					0.935	0.552	0.90
	✓				0.962	0.664	0.94
		✓			0.970	0.672	0.94
			✓		0.940	0.565	0.91
YOLOv8m				✓	0.941	0.566	0.91
		✓	✓		0.977	0.670	0.95
			✓	✓	0.946	0.571	0.92
		✓		✓	0.972	0.671	0.95
		✓	✓	✓	0.980	0.673	0.95

**Table 7:** Ablation Experiments on CSAM Components and CFAE Configuration Choices (M3FD Dataset).

Components	mAP@0.5	mAP@0.5-0.95	Param(M)	GFLOPs
CMADF	0.769	0.491	7.9	21.7
CSAM	0.776	0.512	8.2	22.4
RepVGG	0.762	0.497	3.0	8.4
TOOD	0.721	0.501	3.3	9.1

**Table 8:** Ablation Experiments on CSAM Components and CFAE Configuration Choices (LLVIP Dataset).

Components	mAP@0.5	mAP@0.5-0.95	Param(M)	GFLOPs
CMADF	0.959	0.631	7.9	21.7
CSAM	0.964	0.649	8.2	22.4
RepVGG	0.937	0.557	3.0	8.4
TOOD	0.942	0.561	3.3	9.1

**Fig 13:**
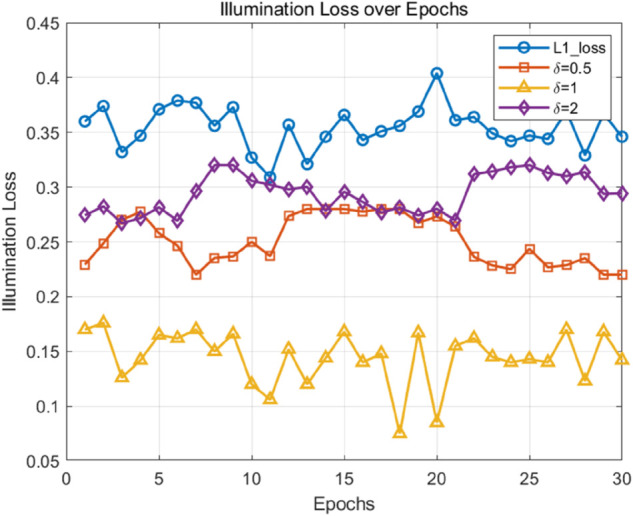
LLVIP dataset illumination loss over epoch.

**Fig 14:**
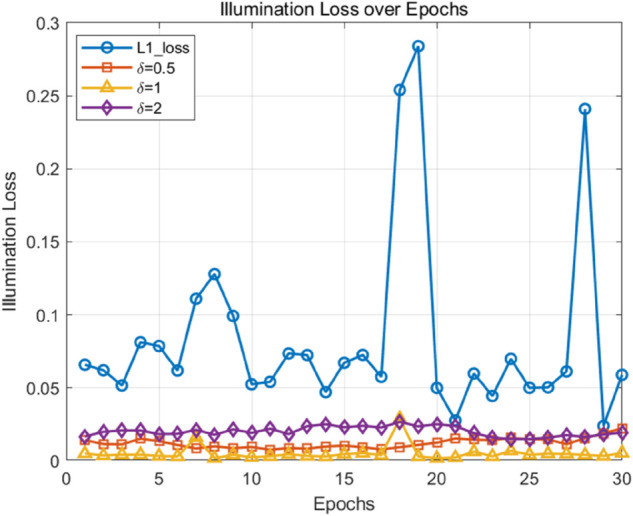
M3FD dataset illumination loss over epoch.

We conducted 30 epochs of image fusion training and evaluation in LLVIP and M3FD dataset to observe the loss curves for δ=0.5,1,2, as well as the original L1 loss. As shown in the Figs 13 and 14, the model achieves the lowest loss when δ=1, which also outperforms the original L1 loss. This demonstrates that δ=1 provides an optimal balance between robustness and sensitivity during training.

The EPIAFusion module shows the most significant improvement, capturing the textural details of objects from visible light and feature information from infrared light, while preserving environmental lighting through light perception. As a result, mAP increased by 1.3% on the M3FD dataset and 3.5% on the LLVIP dataset compared to using only visible light input. The CFAE module enhances the network’s sensitivity to occluded objects by integrating features with distinct receptive fields, resulting in mAP improvement of 1.8% and 0.5%. The SAFDH module, through task alignment and shared convolutions, not only reduced the number of parameters but also elevated the mAP by 1.3% and 0.6%, respectively. Ablation studies confirm that the proposed modules have significantly improved average accuracy on both the M3FD and LLVIP datasets compared to the benchmark model.

### Result visualization

#### M3FD visualization analysis.

**Fig 15:**
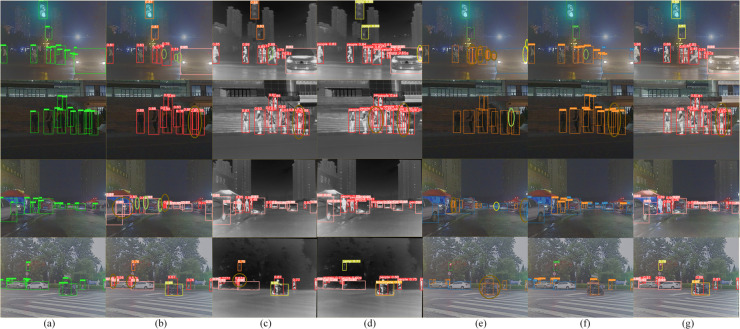
Partial display of detection results on the M3FD dataset (a) M3FD dataset ground truth (b) the prediction results of YOLOv8 using the visible light Input (c) the prediction results of YOLOv8 using the infrared light Input (d) the prediction results of YOLO-MIF (e) the prediction results of ICAFusion (f) the prediction results of RGBT-YOLO (g) the prediction results of FID-YOLO.

Fig 15 shows some prediction results of the models that performed well on the M3FD dataset. Undetected objects are marked with yellow circles, while false positives are indicated by green circles, and multiple detections are highlighted with gold circles. In the first set of images, YOLOv8 shows a significant number of missed detections in both visible and infrared light, while our model and YOLO-MIF perform better. The second and third sets of images were captured under nighttime obstruction conditions, where FID-YOLO exhibits higher confidence with no missed detections or false positives. In the final set of images, which involves detecting small, occluded pedestrian objects, YOLOv8, YOLO-MIF, and ICAFusion all experience issues with multiple detections, while our model successfully detects all targets, demonstrating its superior detection capability in complex environments.

#### LLVIP visualization analysis.

**Fig 16:**
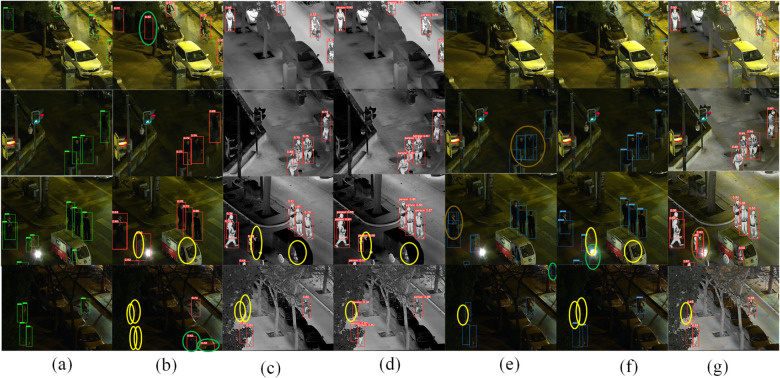
Partial display of detection results on the LLVIP dataset (a)LLVIP dataset ground truth (b) the prediction results of YOLOv8 using the visible light Input (c) the prediction results of YOLOv8 using the infrared light Input (d) the prediction results of YOLO-MIF (e) the prediction results of ICAFusion (f) the prediction results of RGBT-YOLO (g) the prediction results of FID-YOLO.

Fig 16 shows a subset of prediction results for the well-performing models on the LLVIP dataset. False positives are indicated by orange circular markers, while missed detections are represented by green circles and multiple detections are highlighted with gold circles. In the initial set of imagery, YOLOv8 was unable to accurately identify the object features due to the constraints of the nocturnal environment, resulting in false detections. In the second set of images, other models demonstrated good detection performance; however, there were differences in overall confidence compared to FID-YOLO, and the ICAFusion model produced duplicate detections. The third set of photographs revealed that other models failed to identify certain objects, particularly smaller items such as courier vehicles and motorcycles under visible light and infrared conditions. Although our model produced a single duplicate detection, its overall performance remained superior to that of the other models. In the final set of images, our model still maintained good detection performance, with only one missed detection, while the other models exhibited significant issues with both missed and false detections. This demonstrates that our model has better detection capabilities under low-light conditions compared to other models.

**Fig 17:**
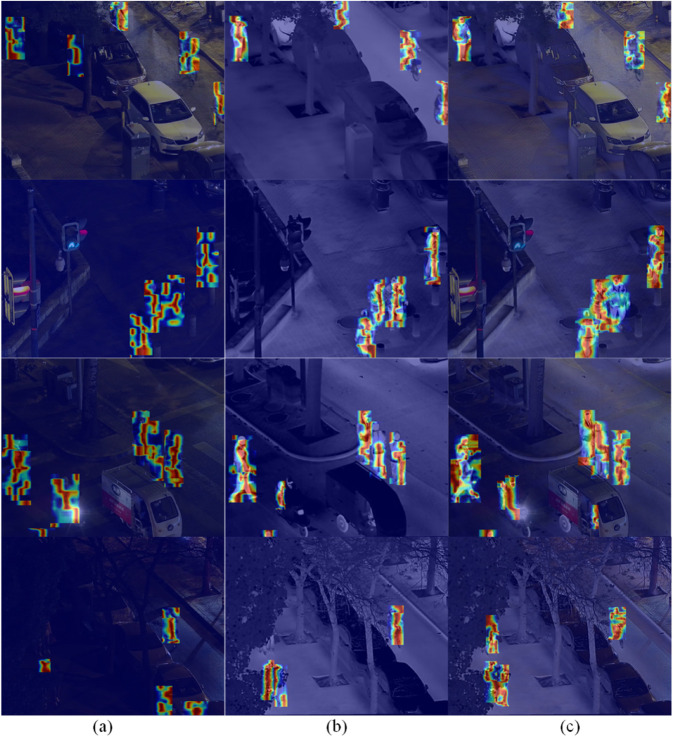
Partial detection effect heatmaps on the LLVIP dataset (a) YOLOv8 visible light input (b) YOLOv8 infrared light input (c) FID-YOLO.

Fig 17 shows the object feature heatmaps on the LLVIP dataset. Compared to YOLOv8, the heatmaps of FID-YOLO more accurately align with the detected objects. In the initial set of imagery, under poor lighting conditions, the network’s output focuses on the background and other irrelevant areas, while FID-YOLO shows greater focus on the relevant objects.

## Conclusions

This study introduces FID-YOLO, an object detection approach that integrate visible and infrared imagery. FID-YOLO integrates an advanced image fusion technique with the YOLOv8 architecture, creating a synergy of information from both visible and infrared light. The process begins with the EPIAFusion module, which extracts and maps features from infrared and visible light into a unified feature space. Subsequently, the resulting fused image is processed by the adapted YOLOv8 framework. In this framework, the CFAE feature aggregation module integrates features from deeper and shallower layers, enhancing the model’s ability to distinguish occluded pedestrians from their surroundings. Finally, SAFDH captures object characteristics across varying scales, improving the model’s feature representation and addressing the challenge of detecting small objects in low-resolution images. Comparative experiments and ablation studies conducted on the M3FD and LLVIP datasets validate the effectiveness and feasibility of the proposed method for Pedestrian detection.

Although FID-YOLO performs well in pedestrian detection, it has some limitations. Model efficiency (parameters and FLOPs) can be further improved, especially for resource-constrained devices. Fusion may degrade performance in cases of very low-quality infrared images or severe misalignment between modalities. Moreover, the model is trained on well-aligned datasets, limiting generalization to non-aligned scenarios. Future work will focus on lightweight design, adaptive fusion, faster inference, and handling non-aligned data to enhance accuracy, efficiency, and robustness.

## Supporting information

S1 Data Experimental results and evaluation data supporting the findings of this study. (XLSX)
